# Exploring Spatial Influence of Remotely Sensed PM_2.5_ Concentration Using a Developed Deep Convolutional Neural Network Model

**DOI:** 10.3390/ijerph16030454

**Published:** 2019-02-04

**Authors:** Junming Li, Meijun Jin, Honglin Li

**Affiliations:** 1 School of Statistics, Shanxi University of Finance and Economics, Wucheng Road 696, Taiyuan 030006, China; Lijunming_dr@126.com or Lijm@sxufe.edu.cn; 2 College of Architecture and Civil Engineering, Taiyuan University of Technology, Yingze Street 79, Taiyuan 030024, China; 3 Shanxi Centre of Remote Sensing, 136 Street Yingze, Taiyuan 030001, China; Lihonglin_sx@126.com

**Keywords:** spatial influence, PM_2.5_ pollution, deep convolutional network, remote sensing

## Abstract

Currently, more and more remotely sensed data are being accumulated, and the spatial analysis methods for remotely sensed data, especially big data, are desiderating innovation. A deep convolutional network (CNN) model is proposed in this paper for exploiting the spatial influence feature in remotely sensed data. The method was applied in investigating the magnitude of the spatial influence of four factors—population, gross domestic product (GDP), terrain, land-use and land-cover (LULC)—on remotely sensed ${\mathrm{PM}}_{2.5}$ concentration over China. Satisfactory results were produced by the method. It demonstrates that the deep CNN model can be well applied in the field of spatial analysing remotely sensed big data. And the accuracy of the deep CNN is much higher than of geographically weighted regression (GWR) based on comparation. The results showed that population spatial density, GDP spatial density, terrain, and LULC could together determine the spatial distribution of ${\mathrm{PM}}_{2.5}$ annual concentrations with an overall spatial influencing magnitude of 97.85%. Population, GDP, terrain, and LULC have individual spatial influencing magnitudes of 47.12% and 36.13%, 50.07% and 40.91% on ${\mathrm{PM}}_{2.5}$ annual concentrations respectively. Terrain and LULC are the dominating spatial influencing factors, and only these two factors together may approximately determine the spatial pattern of ${\mathrm{PM}}_{2.5}$ annual concentration over China with a high spatial influencing magnitude of 96.65%.

## 1. Introduction

Remote sensing technology has developed rapidly since the 1960s [1], and an abundance of remote sensing data has been accumulated in this 50-year period. Although abundant remotely sensed data have been applied to many fields, such as ecology, environment, geography, etc., the spatial analysis method for remotely sensed lattice data desiderates innovation. A single spatial variable generally has autocorrelation [2] (i.e., spatial dependence [3,4]), and various spatial variables have correlation. Spatial autocorrelations can be analysed with local indicators of the spatial association (LISA) index [5] (e.g., local Moran I [6], local Geary c index [7]). The main objective of spatial analysis is to identify the natural relationships that exist between variables [8,9]. The mainstream classical spatial analysis models, e.g., spatial lag model [10,11], spatial error model [10,11], and Bayesian spatial regression model [12], can only evaluate the overall or average linear correlation feature over a whole study region, neglecting the details of local area. These methods ignore the consequences of spatial heterogeneity [13]. The majority of spatial analysing methods assume stationary space. However, assuming spatial convariance structure to be stationary is not so reasonable [14]. The spatial influencing relationship can better be explored when the analysis is local and more detailed results can be yielded [15]. The inclusion of a spatial heterogeneity resulting from differences in environmental conditions, socioeconomic dynamics, and other factors reinforces the need for more regionalized spatial analyses in exposure assessment and public health [16]. Although, the geographically weighted regression (GWR) [17] method considers local details; however, it can only describe a linear or simple non-linear spatial influencing relationship. In the era of big data, the need for developing advanced spatial analysis methods (e.g., machine learning methods) for remotely sensed data is urgent.

Previously, there are several studies that have applied machine learning methods to address the influencing factors on ${\mathrm{PM}}_{2.5}$ concentrations. Zheng et al. [18] used traditional artificial neural networks to model spatial correlation between Beijing’s air qualities and influencing factors, e.g., meteorology, traffic flow, human mobility. Yan et al. [19] predicted the daily average PM_2.5_ concentration in Nanjing, Beijing, and Sanya, combining meteorological and contaminant factors based on the Long Short-Term Memory (LSTM) model. Suleiman et al. [20] presented a machine learning model to predict the traffic-related ${\mathrm{PM}}_{10}$ and ${\mathrm{PM}}_{2.5}$ concentrations from various variables (e.g., traffic variables). Hsieh et al. [21] proposed a semi-supervised learning algorithm to optimize the monitoring locations of air quality in Beijing based on spatial correlation. Certainly, there are some studies which utilized typical methods to investigate the influence of satellite-based PM_2.5_. He et al. [22] used empirical orthogonal function (EOF) to analyse the relationship between remotely sensed ${\mathrm{PM}}_{2.5}$ and climate circulation transformation in East China. Hajiloo et al. [23] employed geographical weight regression (GWR) to investigate impact of meteorological and environmental parameters on PM2.5 concentrations in Tehran, Iran. Yang et al. [24] quantified the influence of natural and socioeconomic factors on ${\mathrm{PM}}_{2.5}$ pollution using the GeoDetector model [25,26]. 

This study proposed a spatial analysis method that exploits the spatial influencing feature of remotely sensed data based on the deep CNN. CNN is a mainstream deep learning method and can effectively extract the feature representations from a large number of images [27] and object detection [28]. Some researchers have applied deep CNN in remote sensing classification. Q. Zou et al. [29] and Zhao et al. [30] proposed a DBN method for high-solution satellite imagery classification. H. Liang and Q. Li, C. Tao et al., and F.P.S. Luus et al. [31], Nogueira et al. [32], Volpi et al. [33], Chen et al. [34], have employed deep CNN in hyperspectral imagery classification or feature extraction. Some researchers have also employed deep CNN in synthetic aperture radar (SAR) image classification, e.g., Du et al. [35] and Geng et al. [36]. To our knowledge, the research applying deep CNN into spatial influencing of remotely sensed lattice data is very rare. This study aimed to present a deep CNN model exploiting the magnitude of spatial influence of four factors—population, gross domestic product (GDP), terrain, and land-use and land-cover (LULC)—to remotely sense the annual mean concentration of ${\mathrm{PM}}_{2.5}$ over China. This model not only considers local spatial heterogeneity but also has super nonlinear fitting ability. Therefore, the presented model is rooted in a deep learning framework and may reduce uncertainty of the results obtained from a simplistic correlation analysis or simple regression model, therefore giving better information to decision makers of public health.

## 2. Materials and Methodology

### 2.1. Materials

The materials used in this research contain five types of data: remotely sensed ${\mathrm{PM}}_{2.5}$ concentration, population spatial distribution density, GDP spatial distribution density, terrain data, and LULC in China. The remotely sensed PM_2.5_ annual concentration dataset in 2010 was produced by the Atmospheric Physics Institute of Dalhousie University in Canada [37] with a resolution of 0.1°×0.1°. The population density data in this paper are cited in the Gridded Population of the World (GPW), data of the UN-Adjust Population Density-v4 [38], published by a data centre in NASA’s Earth Observing System Data and Information System (EOSDIS), with a resolution of 30′ × 30′. GDP spatial distribution density, terrain data, and LULC datasets were drawn from the Resources and Environmental Science Centre of the Chinese Academy of Sciences (http://www.resdc.cn). All above-mentioned data were projected by Albers Conic Equal Area with WGS-84 datum, and the resolution was unified to 10 km×10 km.

### 2.2. Methodology

The methodology in this paper consists of two modules: processing geospatial data and structuring the deep CNN model. The purpose of the former is to establish the dataset for the deep CNN model. The deep CNN model undertakes the mission of fitting the complex function of spatial correlation relationship.

#### 2.2.1. Processing Geospatial Data

**Figure 1:**
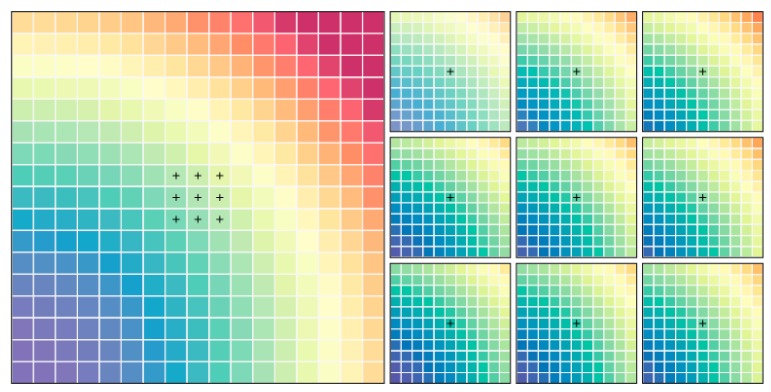
Illustrating 5-order shape of the extent of spatial correlation.

The deep CNN method is usually applied in image identification or classification, not directly transplanted into analysing geospatial data. In the geospatial issue, spatial correlation and geographical attribute need to be considered. Hence, geospatial data require technical processing to match the deep CNN model structure. The four influencing factors generate inputs. Each pixel location contains ${\mathrm{PM}}_{2.5}$ concentrations as output and four influencing factors. In view of spatial correlation, the pixel location and the surrounding locations should be considered. The deep CNN model has the ability of processing big data; therefore the order of spatial correlation can be amplified. In this paper, the order of spatial correlation adopts n-order shape, (2n+1)×(2n+1) pixels (n=1,2,…). Figure 1 shows an illustration of 5-order shape of the spatial correlation extent, including 11×11 pixels. Subsequently, it can extract the corresponding four sets of influencing factor attribute data for a pixel location. Each dataset of influencing factors comprises the corresponding values of the surrounding (2n+1)×(2n+1) pixels. In short, the ${\mathrm{PM}}_{2.5}$ annual concentration of a pixel location is affected by the four influencing factors of its own and the surrounding n-order spatial correlation extent, (2n+1)×(2n+1) pixels. The mathematic form can be expressed as follows:

(1) $$C_{i}^{\left(PM_{2.5}\right)}=F\left(Pop_{i|\left(2n+1\right)\times \left(2n+1\right)},GDP_{i|\left(2n+1\right)\times \left(2n+1\right)},Ter_{i|\left(2n+1\right)\times \left(2n+1\right)},LULC_{i|\left(2n+1\right)\times \left(2n+1\right)}\right)+\xi_{i}$$

where $C_{i}^{\left(PM_{2.5}\right)}$ is the ${\mathrm{PM}}_{2.5}$ annual concentration of the *i*-th pixel, $Pop_{i|\left(2n+1\right)\times \left(2n+1\right)}$, $GDP_{i|\left(2n+1\right)\times \left(2n+1\right)}$,$\;Ter_{i|\left(2n+1\right)\times \left(2n+1\right)}$,$\;LULC_{i|\left(2n+1\right)\times \left(2n+1\right)}$ represent the four influencing factor attribute values of the *i*-th pixel and its surrounding (2n+1)×(2n+1) pixels, and $\xi_{i}$ represents the error. The spatial influencing function F(.) can be learned by the deep CNN model.

#### 2.2.2. A Developed Deep Convolutional Neural Network Model 

CNN contains two categories of cells in the visual cortex, simple cells which exploit local features and complex cells which “pool” (e.g., maximizing, averaging) the outputs of simple cells within a neighbourhood. The structure of CNN model which has two special aspects of local connections and sharing weights is different from general deep learning models. A complete deep CNN stack three types of layers, convolutional layers, pooling layers, and full connected layers. 

**Figure 2:**
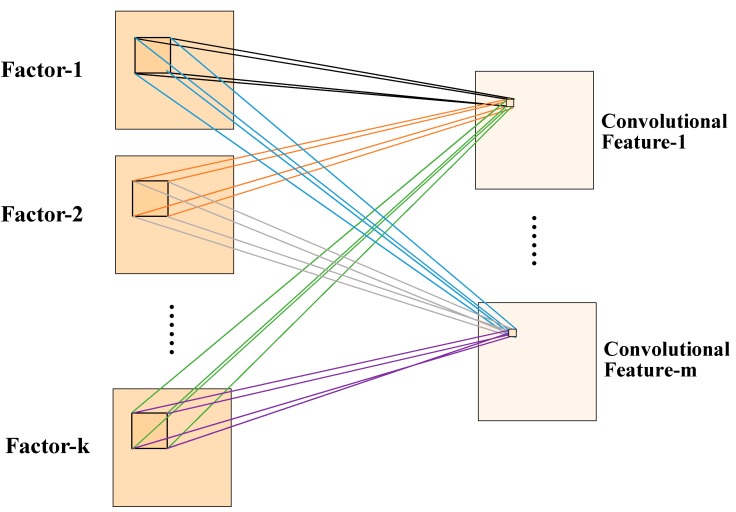
The illustration of 3-D convolution with m (m = 1, 2, …) filters and k (k = 1, 2, …) convolution kernels, the weights are color-coded.

The commonly used CNNs are 2-Dimensional CNN and 3- Dimensional (3-D) CNN. Figure 2 shows a 3-D CNN illustration with m (m = 1, 2, …) filters and k (k = 1, 2, …) convolution kernels. The value of a neuron $S_{\mathrm{ij}}^{xyz}$ at position (x,y,z) of the *j-*th convolutional feature in the *i-*th layer can be expressed as follows [34]:

(2) $$S_{\mathrm{ij}}^{xyz}=C\left(\underset{m}{\sum }\overset{P_{i}-1}{\underset{p=0}{\sum }}\overset{Q_{i}-1}{\underset{q=0}{\sum }}\overset{K_{i}-1}{\underset{k=0}{\sum }}w_{ijm}^{pqk}S_{\left(i-1\right)m}^{\left(x+p\right)\left(y+q\right)\left(z+k\right)}+b_{ij}\right)$$

where *m* indexes the convolutional feature in the (i−1)th layer connected to the *j-*th convolutional feature, and $P_{i}$ and $Q_{i}$ are the height and the width of the convolutional kernel. $K_{i}$ is the size of the spatial influencing factors, $w_{ijm}^{pqk}$ is the value of position (p,q,k) connected to the *m-*th convolutional feature, and $b_{ij}$ is the bias of the *j-*th convolutional feature in the *i-*th layer.

**Figure 3:**
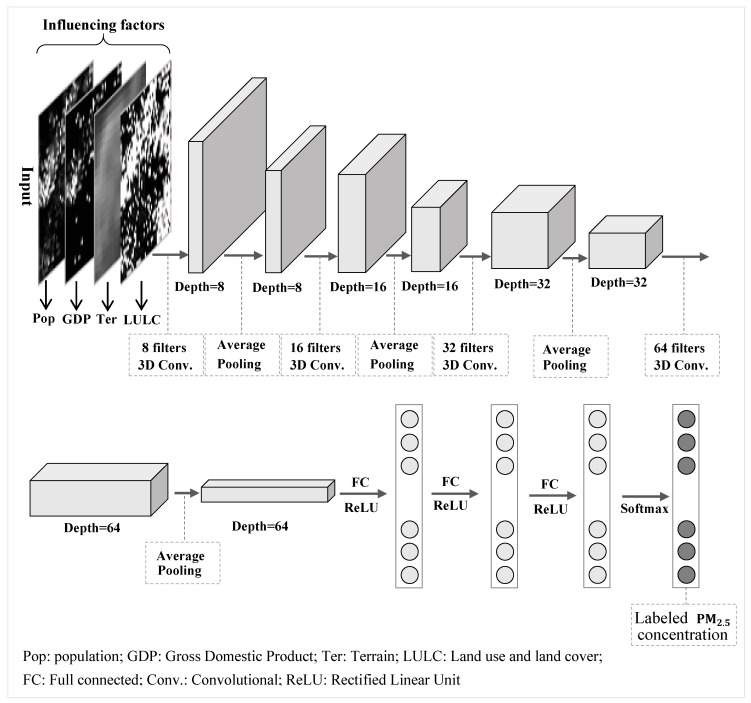
Illustration of the presented deep CNN model architecture exploiting spatial influencing feature of remotely sensed ${\mathrm{PM}}_{2.5}$ concentration, including four convolutional layers, four polling layers, and three hidden layers, the first layer is input containing four influencing factors’ values on a pixel, the output layer with 11 neurons consisting of 11 categories of ${\mathrm{PM}}_{2.5}$ annual concentrations on the pixel location in the middle.

This paper designs a deep 3-D CNN model for exploiting spatial influencing feature of remotely sensed data. Figure 3 illustrates the presented deep CNN model architecture which contains including four convolutional layers, four polling layers, and three hidden layers. And the activation function for hidden layer adopted the Rectified Linear Unit (ReLU) function. The pooling mode employed average mode. The batch normalization was set in each layer except for the output layer. The dropout ratio and learning ratio were set as 25% and 1%, respectively. The dimension of the pre-processed input neural layer is (2n+1)×(2n+1)×4, including four sets of influencing factors with the *i-*th pixel and its surrounding (2n+1)×(2n+1) pixels. Table 1 lists the experimental results when the spatial correlation parameter n was assigned various values. It shows that the validation accuracy reaches the highest when the spatial correlation parameter, n, is taken 9, although the training accuracy is improved along with the increase of the parameter, n. Considering that the validation accuracy is better indicator representing the accuracy of a model. Hence, the spatial correlation parameter, n, is assigned with nine. Then the input layer contains 19 × 19 × 4 neurons with the four factors’ attribute value. The number of the output neuron is 11, labelled by ${\mathrm{PM}}_{2.5}$ annual concentration with 11 categories: <10 $\mu g/m^{3}$, $10\sim 20\;\mu g/m^{3}$, $20\sim 30\;\mu g/m^{3}$, $30\sim 40\;\mu g/m^{3}$, $40\sim 50\;\mu g/m^{3}$, $50\sim 60\;\mu g/m^{3}$, $60\sim 70\;\mu g/m^{3}$, $70\sim 80\;\mu g/m^{3}$, $80\sim 90\;\mu g/m^{3}$, $90\sim 100\;\mu g/m^{3}$, >100 $\mu g/m^{3}$.

## 3. Results

The remotely sensed ${\mathrm{PM}}_{2.5}$ annual concentration and influencing factors possessed 96,337 pixels, among which, 86,903 pixels (accounting for the ratio of 90%) were used for deep learning, and the remaining 9434 pixels (accounting for 10%) were reserved for validation. Training accuracy is defined as the accuracy applied to the training data (i.e., 86,903 pixels), while validation accuracy is the accuracy for the remaining data (i.e., 9434 pixels), and estimated accuracy is the accuracy for the total data (i.e., 96,337 pixels). To investigate the integrated and respective spatial influence of the four various factors, we exploited the congregate magnitude of spatial influence from the four factors and the separate influencing magnitude from one or two factors. 

### 3.1. Integrated Spatial Influencing Feature

**Figure 4:**
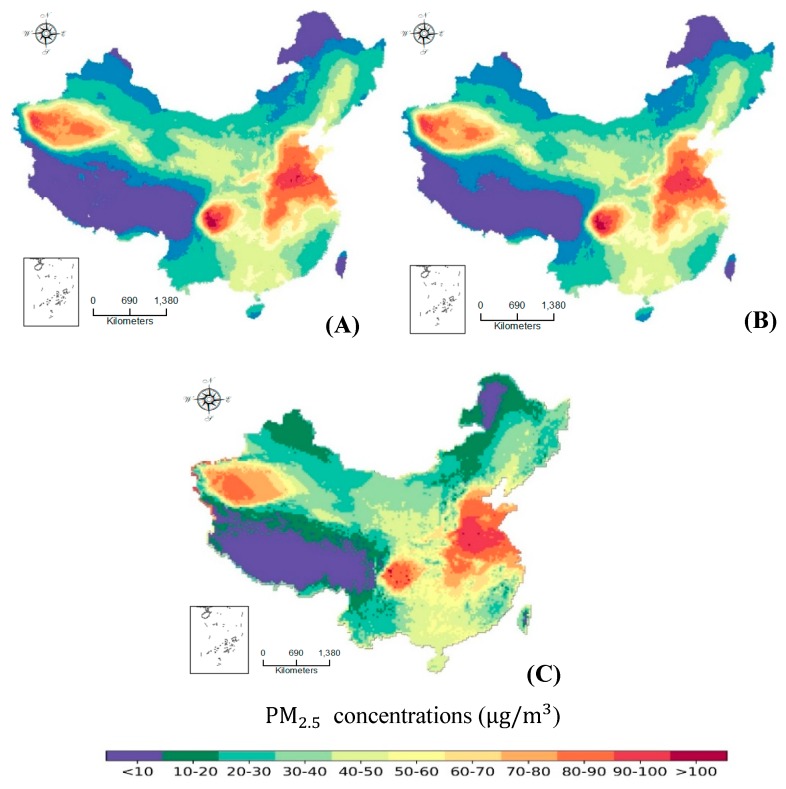
Original (**A**), estimated spatial distribution of ${\mathrm{PM}}_{2.5}$ annual mean concentrations in 2010 (**B**) by the deep CNN model and (**C**) Geographic Weighted Regression (GWR) model, with the four influencing factors (population spatial density, GDP spatial density, terrain, and LULC) over China in 2010.

If the four impact factors were all fed into the input layer, after 1000 epochs of learning, the training accuracy of 86,903 pixels were 98.71%, and the validation accuracy of the remaining 9434 pixels reached 93.29%. Figure 4 illustrates the spatial distribution of the original and estimated ${\mathrm{PM}}_{2.5}$ annual concentration of the total 96,337 pixels using the trained deep learning model fed with the four influencing factors. 

The estimated spatial distribution of ${\mathrm{PM}}_{2.5}$ annual concentration was nearly the same, except for very few pixel locations. It indicated that the four factors (population spatial density, GDP spatial density, terrain, and LULC) can almost determine the ${\mathrm{PM}}_{2.5}$ annual concentration. Furthermore, Table 2 listed the corresponding confusion matrix between the original and estimated ${\mathrm{PM}}_{2.5}$ annual concentration of the total 96,337 pixel locations by the four factors using the trained deep CNN model. The result showed that although there are some incorrect estimated pixel values which were close to the correct values, that is an obvious narrow diagonal band. The overall estimated accuracy is 97.85%. The estimated accuracy of the first category of ${\mathrm{PM}}_{2.5}$ annual concentration, <10 $\mu g/m^{3},$ reaches a maximum of 99.38%. The minimum and the second minimum predicted accuracies are 90.81% and 95.48% respectively, occurring on the eighth and eleventh category of $70\sim 80\;\mu g/m^{3}$ and $90\sim 100\;\mu g/m^{3}$. The estimated accuracy can be regarded as the spatial influencing magnitude of the influencing factors on ${\mathrm{PM}}_{2.5}$ annual concentration. A high estimated accuracy reflects directly a high spatial influencing feature. The results show that there is a strong correlation between ${\mathrm{PM}}_{2.5}$ annual concentration and the four factors. Especially while the trained deep CNN evaluated the total 96,337 pixels, the overall estimated accuracy has reached up to 97.85%, indicating the spatial influencing magnitude of the four factors on ${\mathrm{PM}}_{2.5}$ annual concentration.

### 3.2. Single Spatial Influencing Feature

The spatial influencing magnitude of the single factor can be measured by the deep CNN model proposed in this paper. We have implemented other deep CNNs whose input layer contains 19×19 neurons with a single factor attribute value; the other parameters are the same as above. After 1000 epochs of learning, the training accuracy and validation accuracy of population spatial density and GDP spatial density were 47.12% and 36.13%, 50.07% and 40.91%. Furthermore, the results show that ${\mathrm{PM}}_{2.5}$ annual concentration has strong spatial correlation with terrain or LULC, as the validation accuracies of terrain and LULC were up to 83.17% and 72.37%. The result showed that although the overall estimated accuracies of population and GDP over China were relatively low, the two factors could have determined the severe ${\mathrm{PM}}_{2.5}$ polluted region. Furthermore, the result indicated that terrain and LULC are the main spatial influencing factors on ${\mathrm{PM}}_{2.5}$ annual concentration over China.

In addition, we also have implemented the deep CNN with an input layer containing 19×19×2 neurons describing terrain and LULC. The learning result shows that the training accuracy and validation accuracy of the two factors, terrain and LULC, were up to 90.69% and 87.95%. Table 3 listed the corresponding confusion matrix between the original and estimated ${\mathrm{PM}}_{2.5}$ annual concentration produced by the trained deep CNN fed by terrain and LULC data on the total 96,337 pixel locations. Except for the eleventh category (>100 $\mu g/m^{3}$) of ${\mathrm{PM}}_{2.5}$ annual concentration, the other ten categories’ estimated accuracies are more than 91%. Furthermore, the overall estimated precision can reach up to 96.65%. 

### 3.3. Comparation with the GWR Prediction

To verify the advantage of the deep CNN model presented in this paper, we conducted the GWR in the same dataset. Figure 4C is the estimated spatial distribution of ${\mathrm{PM}}_{2.5}$ annual concentrations over China in 2010 by the GWR model. It can be seen that the GWR estimated results have obvious bias comparing with the origin data (Figure 4). Furthermore, the lowest and highest ${\mathrm{PM}}_{2.5}$ concentrations were particularly misestimated by the GWR model. And the overall estimated accuracy was 72.81% which is more less than the estimated accuracy of the deep CNN model, 97.85%. Comparing the Figure 4B,C, it indicated that although the overall spatial structure estimated by the GWR is generally similar with the origin spatial structure of ${\mathrm{PM}}_{2.5}$ annual concentrations, there were some deviations in detail. The cause of the difference of the two models could be that, the deep CNN model has super strong non-linear fitting ability which can train very complicated non-linear function, however, the GWR is still a linear regression model which cannot catch complicated non-linear variation effects. Inaccurate correlativity between ${\mathrm{PM}}_{2.5}$ concentration and other influencing factors could lead to biased public policies. Scientific public policy-making need more fine and accurate analysing evidences.

## 4. Discussion 

This study proposed a deep CNN model to exploit spatial influencing magnitude for the annual mean concentration of remotely sensed ${\mathrm{PM}}_{2.5}$ over China. In consideration of the influencing mechanism and the availability of the dataset, this study investigated the spatial influence of the four factors (population, GDP, terrain, and LULC) on the annual concentration of ${\mathrm{PM}}_{2.5}$ over China. The influencing factors of ${\mathrm{PM}}_{2.5}$ pollution are known to include natural and anthropogenic activities [39]. Among the four factors selected for this paper, terrain represented natural elements, population and GDP reflected anthropogenic activities, and LULC could be regarded as a mixture of natural and anthropogenic activities. The presented deep CNN method fully considered the local spatial heterogeneity, and a wider spatial correlated scope could be considered by more than one-order shape extent, which benefited from the strong ability of the deep CNN to process big data. 

This paper bridged the gap between spatial analysis and deep CNN technology with the idea of reprocessing or reorganizing remotely sensed data for deep CNN input. The deep CNN method was commonly used to extract the feature representations from a mass of labelled images [27,28]. As aforesaid, few researchers applied the deep CNN model when analysing spatial influence of multiple variables. From a different view, combining a geospatial reprocess, this study designed a 3D deep CNN structure in which the input and output neurons were influencing factors and ${\mathrm{PM}}_{2.5}$ concentration, respectively. The strong non-linear function fitting ability of a deep CNN model could then detect complicated non-linear spatial influencing effect, and the deep CNN model might consider local spatial heterogeneity. From the results, the developed deep CNN model can fully consider spatial relationship and can calculate on each pixel location. Hence, the results can effectively describe the spatial influencing feature on every pixel location. Although the GWR method can also investigate the local correlation on each pixel location, only a linear or simple non-linear regression can be implemented, and the capability of processing big data is not very strong. From the above, the deep CNN model can not only process big data well but can also fit or learn very complicated correlativity. 

This paper demonstrated that the deep CNN technology could be applied in exploiting the spatial influence feature of geospatial or remotely sensed data, and its advantages could be fully performed. The spatial influencing magnitude of the four factors on the annual concentration of ${\mathrm{PM}}_{2.5}$ was investigated employing the presented deep CNN model. This model was not only used in exploring spatial influence of remotely sensed ${\mathrm{PM}}_{2.5}$ concentration, but also in other fields, such as detecting risk factors of some kind of epidemic based on remotely sensed data. Through the model, the risk level of risk factors in public health could be quantificationally assessed. In other words, the developed deep CNN model has the potential to expand the field of spatial analysis of remotely sensed lattice data. Despite all this, this research has some limitations. Firstly, the spatial dependent variable, ${\mathrm{PM}}_{2.5}$ annual concentration, is classified into 11 categories, not as a continuous variable. Secondly, the deep CNN model can learn a very complicated function structure, but the mathematical mechanism is currently not clear, namely mysterious “black boxes” [40], and it is difficult to explain in a geographical process.

## 5. Conclusions

Population spatial density, GDP spatial density, terrain, and LULC can almost determine the spatial pattern of ${\mathrm{PM}}_{2.5}$ annual concentration with an overall estimated precision of 97.85% over China. Furthermore, terrain and LULC are the main spatial influencing factors on ${\mathrm{PM}}_{2.5}$ annual concentration among the four factors. And the overall spatial influencing magnitude of the two factors, terrain and LULC, reached up to 96.65%, nearly equal to all four factors’ spatial influencing magnitude on ${\mathrm{PM}}_{2.5}$ annual concentration.

## Figures and Tables

**Table 1:** Experimental results of the training and validation accuracy of the deep 3-D CNN model when the spatial correlation parameter, n, is assigned various values

Spatial Correlation Parameter, n	Training Accuracy	Validation Accuracy
1	67.94%	80.17%
2	77.71%	82.37%
3	88.08%	86.11%
4	92.01%	90.50%
5	94.51%	91.83%
6	96.53%	92.14%
7	97.35%	92.90%
8	98.30%	92.46%
9	98.71%	93.29%
10	98.87%	92.40%
11	99.29%	93.28%
12	99.53%	93.25%

**Table 2:** The confusion matrix of the original vs. estimated ${\mathrm{PM}}_{2.5}$ annual concentrations by the deep CNN model fed by the four influencing factor data: population, GDP, terrain, and LULC.

96,337 Pixels		Original $PM_{2.5}$ Annual Concentration ($\mu g/m^{3}$)										
		<10	10~20	20~30	30~40	40~50	50~60	60~70	70~80	80~90	90~100	>100
Estimated ${\mathrm{PM}}_{2.5}$ annual concentration ($\mu g/m^{3}$)	<10	18,395	337	0	0	0	0	0	0	0	0	0
	10~20	112	11,792	70	3	0	1	0	0	0	0	0
	20~30	2	83	21,891	86	12	1	0	2	0	0	0
	30~40	0	3	101	10,804	89	3	0	0	0	0	0
	40~50	0	6	18	115	13,971	60	5	0	0	0	0
	50~60	0	0	1	5	62	4103	57	4	0	0	0
	60~70	0	0	0	0	0	49	3650	62	0	0	0
	70~80	0	0	2	0	0	1	142	3598	159	0	0
	80~90	0	0	0	0	0	0	14	296	4563	60	0
	90~100	0	0	0	0	0	0	0	0	38	1332	7
	>100	0	0	0	0	0	0	0	0	1	3	166
	Accuracy	99.38%	96.49%	99.13%	98.10%	98.85%	97.27%	94.36%	90.81%	95.84%	95.48%	95.95%

**Table 3:** The confusion matrix of the original vs. estimated ${\mathrm{PM}}_{2.5}$ annual concentrations by the deep CNN model fed by the two influencing factor data, terrain and LULC.

96,337 Pixels		Original $PM_{2.5}$ Annual Concentrations ( $\mu g/m^{3}$)										
		<10	10~20	20~30	30~40	40~50	50~60	60~70	70~80	80~90	90~100	>100
Estimated ${\mathrm{PM}}_{2.5}$ annual concentration ($\mu g/m^{3}$)	<10	17,974	535	0	0	0	0	0	0	0	0	0
	10~20	161	11,775	135	3	0	0	0	0	0	0	0
	20~30	3	129	21,622	129	20	0	1	6	0	0	0
	30~40	1	3	177	10,869	128	4	1	1	2	0	0
	40~50	1	1	27	181	13,991	90	8	1	8	0	0
	50~60	0	0	2	3	81	4099	93	3	1	0	0
	60~70	0	0	4	1	5	75	3491	75	1	1	0
	70~80	0	0	1	0	1	1	181	3395	193	1	0
	80~90	0	0	1	0	2	0	17	462	4445	108	0
	90~100	0	0	0	0	5	0	5	4	101	1290	40
	>100	0	0	0	0	0	0	0	0	0	4	158
	Accuracy	99.08%	94.63%	98.42%	97.17%	98.30%	96.02%	91.94%	86.01%	93.56%	91.88%	79.80%
